# Behavioral and Demographic Profiles of HIV Transmission and Exposure Networks in Florida: Network Analysis of HIV Contact Tracing Data

**DOI:** 10.2196/65573

**Published:** 2025-06-25

**Authors:** Yiyang Liu, Christina Parisi, Chaoyue Sun, Rebecca Fisk-Hoffman, Marco Salemi, Diego Viteri, Brandi Danforth, Mattia Prosperi, Simone Marini

**Affiliations:** 1 Department of Epidemiology, College of Public Health and Health Professions and College of Medicine, University of Florida, 2004 Mowry Road, Gainesville, FL, 32610, United States, 1 (352) 273-5468; 2 Department of Electrical and Computer Engineering, College of Engineering, University of Florida, Gainesville, FL, United States; 3 Department of Pathology, Immunology and Laboratory Medicine, College of Medicine, University of Florida, Gainesville, FL, United States; 4 Emerging Pathogens Institute, University of Florida, Gainesville, FL, United States; 5 STD Section, Florida Department of Health, Tallahassee, FL, United States

**Keywords:** population health, demographic-behavioral profiles, HIV, HIV prevention, Florida, Surveillance Tools and Reporting System, STARS, United States, public health, health informatics, infectious disease

## Abstract

**Background:**

To complete the Ending the HIV Epidemic initiative in areas with high HIV incidence, there needs to be a greater understanding of the demographic, behavioral, and geographic factors that influence the rate of new HIV diagnoses. This information will aid the creation of targeted prevention and intervention efforts.

**Objective:**

This study aims to identify the geographic distribution of risk groups and their role within potential transmission networks in Florida.

**Methods:**

Public data from the Florida Department of Health and behavioral data from the Surveillance Tools and Reporting System between 2012 and 2022 were used in these analyses. We analyzed records as a combination of variables of interest (gender, age, race or ethnicity, and HIV risk group) to create demographic-behavioral profiles (DBPs) that represent the profiles of people newly diagnosed with HIV. We then used the resulting DBPs to characterize Florida counties and HIV coordination areas and calculated the county-to-county and area-to-area rank (Spearman) correlation. We then drew a dendrogram based on the correlation matrix and identified clusters of similar counties and areas. Lastly, network analysis used HIV contact tracing data from the Surveillance Tools and Reporting System to identify HIV transmission and exposure contact networks and characterized large networks by DBPs and geolocation.

**Results:**

We identified 37 DBPs. The largest DBPs were Hispanic and non-Hispanic Black males aged 25‐49 reporting male-to-male sexual contact (n=7539 and n=4329, respectively), non-Hispanic White males aged 25‐49 reporting male-to-male sexual contact (n=4221), and non-Hispanic Black females aged 25‐49 reporting heterosexual contact (n=3371). The state could be broken up generally into 2 transmission and exposure clusters by region: Northwestern or Northern and Central or Southern. We identified several counties with similar DBPs that were not in the same HIV coordination area. A total of 3097 contact networks were identified among 7944 people with HIV contact tracing data. Most (n=2508, 81%) networks involve only 2 people, 11% (n=349) involve 3 people, 7% (n=224) involve 4 to 19 people, and 6 networks involve 20 or more people. As network size increases, the proportion of people within the network who identify as female, non-Hispanic Black, aged older than 50 years, and exposed to HIV via heterosexual contact decreases.

**Conclusions:**

We identified distinct risk groups and clusters of transmission and exposure throughout Florida. These results can help regions identify health disparities and allocate their HIV prevention and intervention resources accordingly. The goal of this work was to highlight areas of need in a high-incidence setting, not to contribute to existing stigma against vulnerable groups, and it is important to consider the ethics and possible harm of advanced methodologies such as contact network analysis when addressing public health problems.

## Introduction

The burden of HIV has long been felt disproportionately across gender, sexual, and racial or ethnic minority groups in the United States. Historically, men who have sex with men (MSM) have had the highest risk of acquiring HIV and made up a majority (69%) of new HIV diagnoses in 2019 [1]. Among MSM, non-Hispanic Black MSM account for a large proportion of new diagnoses. Furthermore, many non-Hispanic Black and Hispanic MSM are unaware of their HIV status, which can lead to further transmission [1]. While the number of new diagnoses among MSM has generally decreased, in 2019 the number of diagnoses among this group aged 55 years or older increased by 5% [1], so there is heterogeneity among these groups by demographic and behavioral factors. Women make up nearly 20% of new HIV diagnoses, and a majority of transmissions among women are due to heterosexual contact (hetero) [1,2]. Among women, nearly 60% of new diagnoses are among non-Hispanic Black women [1,3]. While most transmissions of HIV are due to sexual contact, injection drug use (IDU) accounts for about 10% of new HIV diagnoses [1]. There is great variability in the demographic composition and behavioral risk factors for HIV, and this variability is even more apparent when considering geographic differences.

The Southern United States experiences higher rates of HIV diagnoses and worse HIV-related outcomes compared with the rest of the nation [1,4,5]. Within the South, Florida has consistently been among the states with the highest HIV incidence for the past decade, and ranked third in new diagnoses in 2019 [1]. Florida also experiences high rates of poverty and low rates of health insurance coverage, and has a fluctuating population due to migration, seasonal residents, and tourism. As is the case nationwide, new HIV diagnoses in Florida disproportionately affect Hispanic and non-Hispanic Black MSM and non-Hispanic Black women [6-8]. In 2019, a total of 7 counties in Florida were selected as geographic priority areas in phase 1 of the Ending the HIV Epidemic (EHE) initiative [9,10]. These counties have a high incidence and prevalence of HIV and include some of the most populous in the state: Miami-Dade, Broward, Palm Beach, Orange, Duval, Pinellas, and Hillsborough. The counties highlighted in the EHE initiative are largely urban, however, rural areas in Florida and the rest of the Southern United States have relatively high HIV prevalence and are disproportionately impacted by HIV [11-13]. Therefore, intervention efforts need to extend beyond the 7 selected counties. Compared with urban areas, rural areas of Florida differ demographically, and past research has found that there are demographic differences between rural and urban areas in Florida in terms of those diagnosed with HIV/AIDS [12,14]. There are also different structural and social barriers that can prevent people from receiving HIV care in urban compared with rural areas. Additionally, stigma and discrimination due to HIV and racial or ethnic, gender, and sexual minority groups can vary by location, within groups, and across cultures, and be substantial barriers to knowledge about HIV, engagement in HIV prevention behaviors, and receipt of HIV health care [15].

There are many effective evidence-based strategies for reducing HIV incidence, such as pre-exposure prophylaxis (PrEP) for people in high-risk social networks [16,17]. Additionally, implementing routine screenings among high-risk groups can aid in identifying HIV infection at an earlier stage, which may lead to the timely initiation of treatment and lower the risk of HIV transmission [18-20]. However, the effectiveness of these prevention efforts is limited by resource availability. Further, these interventions must be targeted and require a thorough assessment of the at-risk population. Although the incidence of HIV has gradually declined within Florida and nationwide in the past decade, new and effectively targeted interventions are needed to achieve the EHE goal of reducing HIV diagnoses by 90% by 2030 [9]. The HIV epidemic impacts people from a wide variety of areas and backgrounds, so a one-size-fits-all approach is unlikely to be effective in meeting these goals. There is a need to identify and understand the diversity of factors that can influence the impact of HIV prevention efforts to create informed and impactful interventions.

Past research has identified clusters of HIV risk groups to reduce transmission and inform targeted interventions [21-23]. Predicting the trajectory of HIV transmission clusters is a pivotal step for both prevention and treatment interventions. In this study, we aimed to use a retrospective public health dashboard and surveillance data to characterize people newly diagnosed with HIV by their demographics, behavioral profile, geolocation clusters, and contact network. Additionally, this study serves as proof of concept for leveraging HIV contact tracing data from surveillance tools to enhance insights into HIV transmission dynamics.

## Methods

### Data Source

We downloaded a public dashboard dataset from the Florida Department of Health (FDOH) through publicly available FLHealthCHARTS. The dataset included all documented new HIV diagnoses between January 1, 2012, and June 30, 2022. Records including diagnoses among those aged 12 years or younger were excluded.

The Surveillance Tools and Reporting System (STARS), formerly known as PRISM (Patient Reporting Investigation Surveillance Manager), is managed by the FDOH. STARS contains relational data collected during contact tracing interviews, where people newly diagnosed with HIV identify recent partners, both through sexual contact and needle-sharing, who are at higher risk of HIV. Information on both people with HIV and their reported contacts is recorded in STARS. These contact tracing interviews are conducted by disease intervention specialists (DIS), who also obtain additional information on demographics and risk behaviors. Partners who are identified are then contacted by DIS to encourage HIV testing, and, if positive, are interviewed again to identify contacts. In general, DIS successfully contacted 90% of people newly diagnosed with HIV and contacted 65%‐70% of the named partners in Florida. STARS data elements used in the current analyses include individual ID and ID from their linked partners, demographic information (age, gender, or race or ethnicity), county of residence, and HIV risk information (male-to-male sexual contact [MMSC], hetero, IDU, etc). To extract data from STARS for network analysis, we retained only records for individuals that could be attributed to a single, specific demographic-behavioral profile (DBP; ie, without missing DBP attributes), and had at least 1 connection with another individual that could also be attributed to a single, specific DBP. We filtered out the records reporting modes of exposure not consistent with the DBPs, such as blood transfusions or prenatal exposures. The remaining modes of exposure matched the modes of exposure in the FDOH public dataset. If an individual showed multiple records over time, we used the median age from these records. Unlike the FDOH public dataset, STARS reported combinations of race and ethnicity. To be able to apply the DBPs to STARS, all people who self-identified as Hispanic were classified as Hispanic, regardless of race. Those who identified as non-Hispanic were further classified based on the race reported in STARS.

### Ethical Considerations

The authors abide by the Declaration of Helsinki. This study protocol was approved by the University of Florida’s Institutional Review Board (IRB) and by FDOH’s IRB (protocol #IRB201901041 and #2020‐069, respectively) as exempt. We received data extracts from FDOH’s STARS in a fully deidentified format according to the HIPAA (Health Insurance Portability and Accountability Act).

### Data Availability

The dashboard data used in this study are publicly available at FLHealthCHART.gov. The STARS data are managed by FDOH. Researchers seeking access for replication purposes need to submit a data request to the FDOH (research@flhealth.gov) by state, federal regulations, and required ethical and privacy policies, including IRB approval by FDOH and execution of a data user agreement. Requests are independently reviewed by FDOH.

### Data Analyses

We identified a total of 4 demographic and behavioral variables common in both FDOH public data and STARS and used their combinations to create DBPs. The four variables are (1) gender: male (M), female (F); (2) age: <25, 25‐49, 50+ years; (3) race or ethnicity: Hispanic, non-Hispanic Black, non-Hispanic White, and non-Hispanic other; and (4) HIV risk group (ie, mode of exposure): MMSC, hetero, IDU, MMSC or IDU, and other. We explored the option of extracting DBPs via unsupervised clustering. Both elbow and silhouette analyses indicated 8 as the suggested number of clusters (data not shown). With the number of clusters (k=8), both agglomerative and divisive clustering approaches based on Gower distance split the data into groups perfectly and uniformly characterized by gender and race or ethnicity (ie, clusters where all the members belong to the same combination of gender and race or ethnicity). When using k>8, data were further partitioned by age (data not shown). However, none of the profile identities from the unsupervised clustering methods include the only behavioral factor (ie, HIV risk group), which we believe is important to include as it is a modifiable factor. Therefore, we decided to extract DBPs based on their frequency, as explained above, instead of using unsupervised clustering.

Descriptive analysis was used to demonstrate the frequencies for identified DBPs and our study sample characteristics. Multiple correspondence analysis (MCA) was implemented using the FactoMineR and factoextra libraries in R Studio (R Foundation). MCA is an extension of simple correspondence analysis and is used to summarize and visualize data tables with more than 2 categorical variables. This analysis was used to show a graphical representation of the similarity between identified DBPs.

Additionally, we used the identified DBPs to characterize Florida counties and HIV coordination areas. HIV coordination areas (“areas”) are groups of counties that operate under the leadership of an HIV/AIDS program coordinator who oversees the region’s HIV health care and prevention efforts within the FDOH [24]. Each county and area can be represented by an array of 37 slots, each representing the counts for a specific patient type. To compare counties and areas, we calculated the county-to-county and area-to-area rank (Spearman) correlation. We then drew the dendrogram based on the correlation matrix and found groups of similar counties and areas.

Lastly, we performed network analysis using STARS data based on the DBPs identified. The Leiden algorithm [25] is a network node partitioning method that focuses on identifying clusters characterized by maximized intracluster connections and minimized intercluster connections. In this study, the algorithm was implemented using the *leidenalg* library in Python (Python Software Foundation) [26], with modularity serving as the optimization function and setting the resolution parameter to 0.2 to control cluster counts.

## Results

### Sample Characteristics and Identified DBPs

Sample characteristics are shown in Table 1 stratified by data source. The Florida Chart public data and STARS data show similar sample characteristics: mostly male, aged 25‐49 years, exposed to HIV via MMSC. Non-Hispanic Black is the most common race or ethnicity group in both data sources.

Using the Florida public data, we identified 84 different DBPs among 44,090 people newly diagnosed with HIV between 2012 and 2022. Of these 84 DBPs, 36 cover 97% of the HIV incident cases (42,734/44,090), and each included 100 or more individuals. The remaining DBPs were combined into an “other” group, making 37 DBPs total. These DBPs are reported in Table 2. The results from the MCA, showing a graphical representation of the similarity between these DBPs, are provided in Figure S1 in Multimedia Appendix 1.

**Table 1.** Sociodemographic comparison between the FDOH^a^ public dataset and STARS^b^. Numerical description of the datasets.

	Florida chart public data (n=44,090), n (%)	STARS data (n=7944), n (%)
Gender		
Female	9526 (21.6)	1530 (19.3)
Male	34,564 (74.4)	6414 (80.7)
Race or ethnicity		
Hispanic	14,043 (31.9)	2039 (25.7)
Non-Hispanic Black	18,343 (41.6)	3839 (48.3)
Non-Hispanic White	10,709 (24.3)	1866 (23.5)
Non-Hispanic other	995 (2.3)	200 (2.5)
Age group (years)		
<25	7654 (17.4)	1695 (21.3)
25‐49	26,925 (61.1)	5908 (74.4)
50+	9511 (21.6)	1151 (14.5)
HIV risk group		
Hetero^c^	14,918 (33.8)	2422 (30.5)
MMSC^d^	26,498 (60.1)	4919 (61.9)
MMSC or IDU^e^	898 (2)	291 (3.7)
IDU	1718 (3.9)	312 (3.9)
Other	58 (0.1)	0 (0)

a FDOH: Florida Department of Health.

b STARS: Surveillance Tools and Reporting System.

c Hetero: heterosexual contact.

d MMSC: male-to-male sexual contact.

e IDU: injection drug use.

**Table 2.** Thirty-six DBPs^a^ were extracted from the FDOH^b^ public dataset among people newly diagnosed with HIV between 2012 and 2023, covering 97% of cases. A 37th “other” DBP included the remaining cases.

DBP	Gender	Ethnicity or race	Age group (years)	Exposure	Cases, n
1	Male	Hispanic	25‐49	MMSC^c^	7539
2	Male	Black Non-Hispanic	25‐49	MMSC	4329
3	Male	White Non-Hispanic	25‐49	MMSC	4221
4	Female	Black Non-Hispanic	25‐49	Hetero^d^	3371
5	Male	Black Non-Hispanic	<25	MMSC	3024
6	Male	Black Non-Hispanic	25‐49	Hetero	2204
7	Male	White Non-Hispanic	50+	MMSC	2066
8	Male	Hispanic	<25	MMSC	1744
9	Female	Black Non-Hispanic	50+	Hetero	1652
10	Male	Hispanic	50+	MMSC	1404
11	Male	Black Non-Hispanic	50+	Hetero	1400
12	Female	Hispanic	25‐49	Hetero	939
13	Male	White Non-Hispanic	<25	MMSC	870
14	Male	Hispanic	25‐49	Hetero	831
15	Female	Black Non-Hispanic	<25	Hetero	748
16	Female	White Non-Hispanic	25‐49	Hetero	690
17	Male	Black Non-Hispanic	50+	MMSC	641
18	Male	Other Non-Hispanic	25‐49	MMSC	444
19	Male	White Non-Hispanic	25‐49	Hetero	444
20	Female	Hispanic	50+	Hetero	431
21	Male	Hispanic	50+	Hetero	418
22	Male	White Non-Hispanic	25‐49	MMSC or IDU^e^	391
23	Female	White Non-Hispanic	25‐49	IDU	380
24	Female	White Non-Hispanic	50+	Hetero	377
25	Male	White Non-Hispanic	50+	Hetero	370
26	Male	Black Non-Hispanic	<25	Hetero	333
27	Male	White Non-Hispanic	25‐49	IDU	303
28	Female	Hispanic	<25	Hetero	165
29	Male	Other Non-Hispanic	<25	MMSC	155
30	Female	White Non-Hispanic	<25	Hetero	144
31	Male	Hispanic	25‐49	MMSC or IDU	130
32	Male	Black Non-Hispanic	25‐49	IDU	123
33	Male	Hispanic	25‐49	IDU	122
34	Male	Black Non-Hispanic	50+	IDU	119
35	Male	Black Non-Hispanic	25‐49	MMSC or IDU	111
36	Female	Other Non-Hispanic	25‐49	Hetero	101

a DBP: demographic-behavioral profile.

b FDOH: Florida Department of Health.

c MMSC: male-to-male sexual contact.

d Hetero: heterosexual contact.

e IDU: injection drug use

### Characterization of County and Area Similarity Based on the DBPs

**Figure 1.**
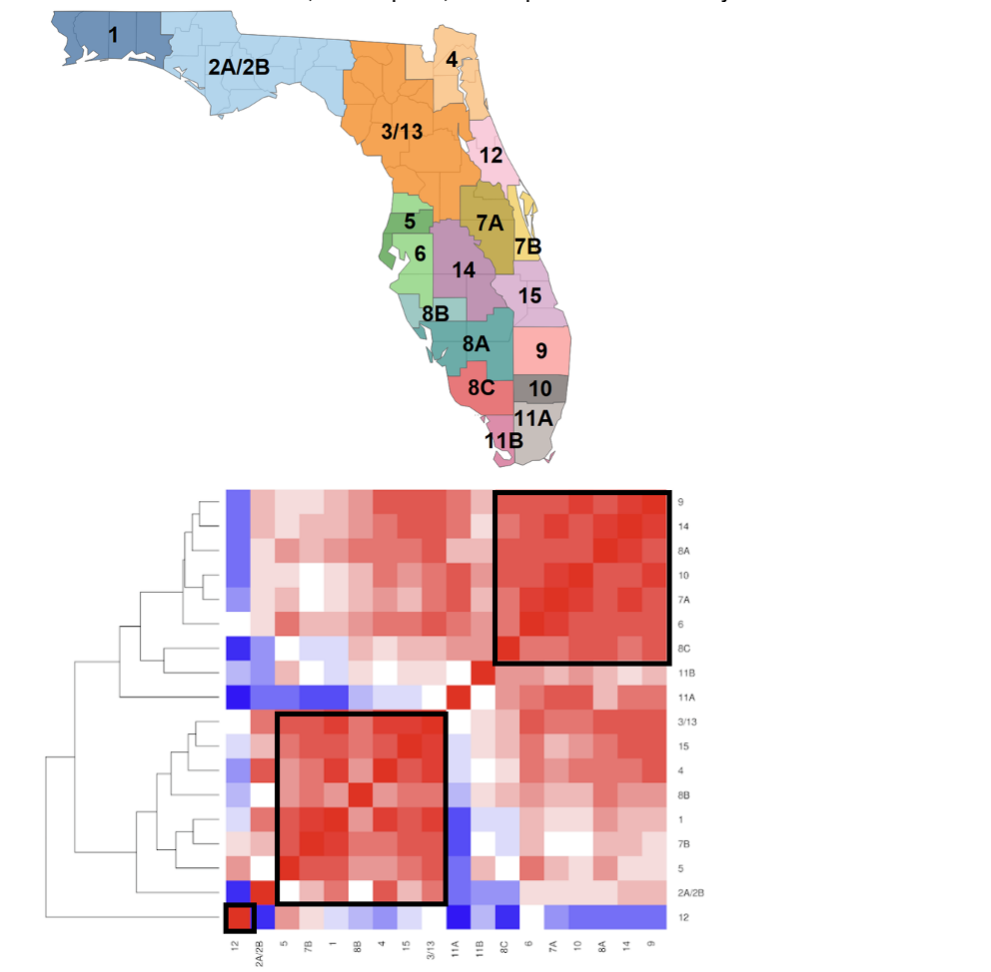
Area similarity based on the sociodemographic profiles. Top panel: map of HIV coordination areas in Florida. Bottom panel: heatmap of the area similarity. In the heatmap, each area is compared to the others per DBPs, that is, with higher similarity shifting toward red, and lower similarity shifting toward blue. Of note, areas are arranged via a data-driven process (hclust) so that similar ones will be closer together, as indicated by the dendrogram on the right. Visual inspection reveals 3 clusters, indicated by the black squares. DBP: demographic-behavioral profile.

The dendrogram showing the correlation matrix between county and HIV coordination areas in Florida using the identified DBPs, is depicted in Figures1  2. Many HIV areas share similar distributions of DBPs and predominant DBP types, and 2 main clusters were identified. The first area cluster included many counties in the western part of Central Florida and Southern Florida (areas 9, 14, 8A, 10, 7A, and 6) and the second represented counties in Northern Florida, the Panhandle (Northwestern Florida), and the east coast of Central Florida (areas 5, 7B, 1 8B, 4, 15, and 3/13). Area 12 (Flagler and Volusia) was dissimilar from most of the other areas. Area 11A (Miami-Dade) was similar to the Central or Southern Florida cluster but very dissimilar from the Northern Florida or Northwestern Florida cluster. Among the counties, rural counties tended to be more similar but there was no clear delineation between rural and urban counties. We identified 6 clusters of varying sizes. The largest included 12 counties: Polk, Palm Beach, Lee, Seminole, Hillsborough, Manatee, Marion, St. Lucie, Indian River, Orange, Broward, and Collier. The next largest cluster included 8 counties: Pinellas, Brevard, Sarasota, Hernando, Bay, Okaloosa, St. Johns, and Pasco. The remaining clusters included between 6 and 2 counties: Jackson, Alachua, Leon, Putnam, and Columbia; Duval, Escambia, and Clay; Flagler and Volusia; and Miami-Dade, Osceola, and Hendry. The Duval, Escambia, and Clay cluster were also similar to the 2 largest clusters, while the 1 containing Miami-Dade also shared many similarities with the counties in the largest cluster.

**Figure 2.**
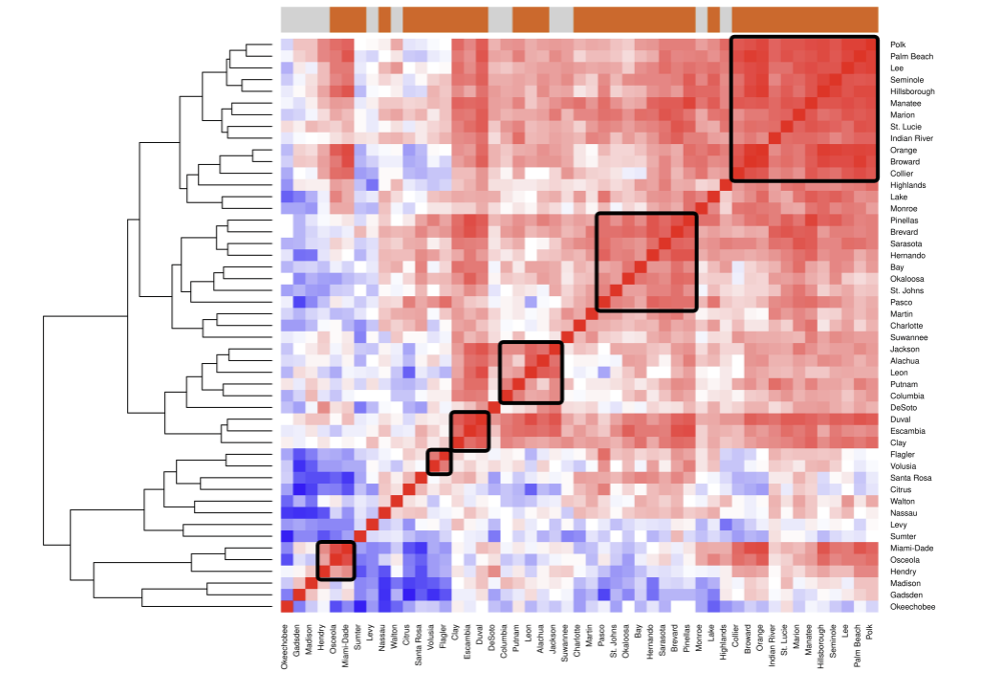
Heatmap of county similarity based on the sociodemographic profiles and by rural or urban (bar on top of figure: orange-urban and gray-rural). Each county is compared to the others per DBPs, that is, with higher similarity shifting toward red, and lower similarity shifting toward blue. Of note, counties are arranged via a data-driven process (hclust) so that similar ones will be closer together, as indicated by the dendrogram on the right. Visual inspection reveals 6 clusters, indicated by the black squares. DBP: demographic-behavioral profile.

### Analysis of HIV Contacts Reveals Mode-of-Exposure Characterizing Both Network Size and Composition

**Figure 3.**
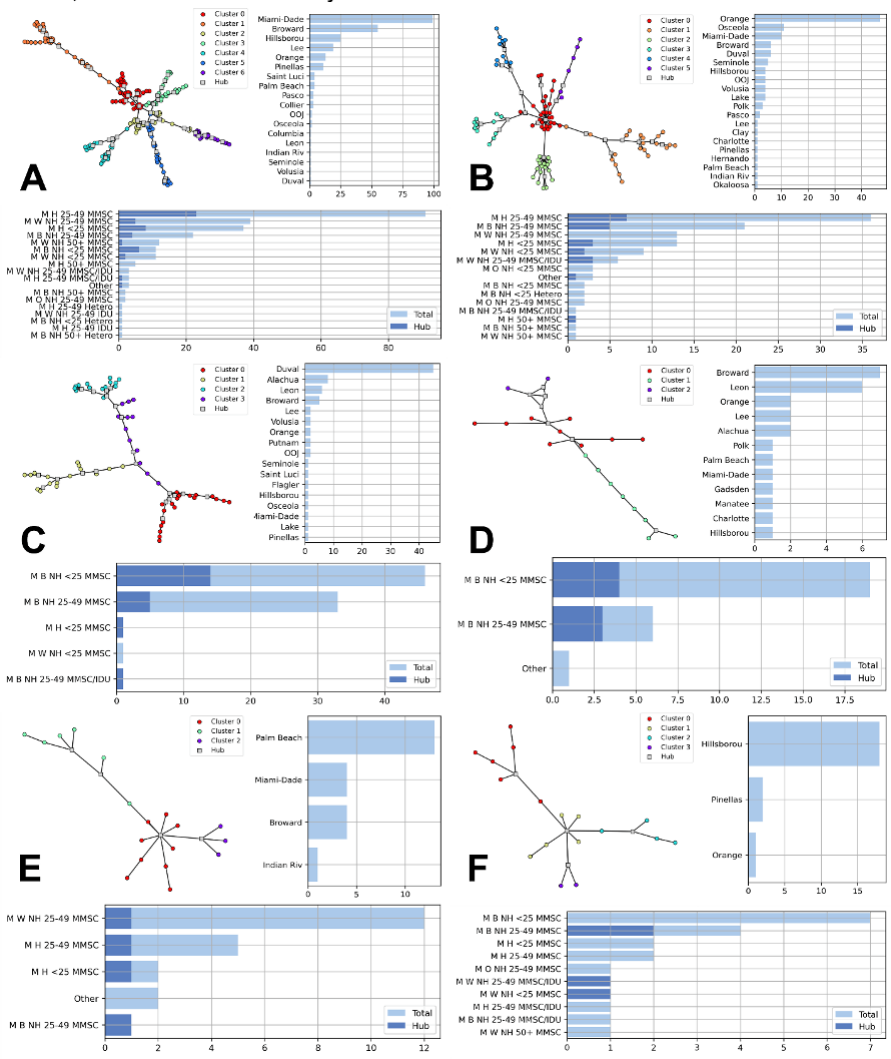
The 6 largest contact networks (20+ nodes), characterized by hub nodes, Leiden Clusters, identified DBPs and county, are represented here ordered on size (largest to smallest), labelled A to F. Each panel represents a network of individuals in our dataset. Nodes (dots) represent individuals, and undirected edges represent connections between individuals based on contact tracing data. Individuals are grouped into clusters using the Leiden algorithm and are colored accordingly—nodes with the same color belong to the same cluster. Notably, this clustering is based solely on the network structure, not on individual characteristics. Some individuals are identified as hubs, defined as nodes with more than 2 connections; these represent individuals with active transmission-related behavior and warrant closer attention. We also provide a graphical description of the aggregated data for each network, shown via bar charts that display the individuals’ counties as well as the identified DBPs within the network, including the fraction of each DBP covered by hub individuals. These results highlight potential differences in transmission patterns. B: Black; DBP: demographic-behavioral profile; H; Hispanic; IDU: injection drug use; MMSC: male-to-male sexual contact; NH: non-Hispanic; O: other; W: White.

STARS represents contact tracing data to create networks of people with HIV and people exposed to HIV. We extracted 7944 individuals from STARS, sharing 4928 connections. The DBP counts in STARS correlate strongly with the counts of the FDOH public dataset (Spearman correlation=0.93). STARS individuals formed 3097 subnetworks (ie, networks that are not connected to one another). Of those, 2518 (~81%) are 2-node networks, 349 (~11%) are 3-node networks, 224 (~7%) networks have 4‐19 nodes, while the remaining 6 networks have 20‐246 nodes (shown in Figure 3). Notably, hetero decreases decisively with the network size, is found more commonly in smaller networks, and its representation decreases with network growth. Table 3 reports the characteristics of the STARS population based on the network size. In the 2 largest contact networks (Figures 3A and 3B), males of Black, Hispanic, and White ethnicities aged 25‐49 years predominantly constitute both networks. Interestingly, when focusing on hub nodes (nodes with more than 2 links in the network), aside from the dominant groups, young Hispanic and Black males younger than the age of 25 years also comprise a significant portion.

**Table 3.** Characteristics of contact networks by number of nodes. Numerical description of extracted networks. For the first 2 rows, the row percentages were calculated. For the rest of the rows, column percentages were calculated.

	Values, n (%)	Values, n (%)	Values, n (%)	Values, n (%)
Number of nodes^a^	2	3	4 to 19	20 to 246
Total networks	2518 (81)	349 (11)	224 (7)	6 (0.2)
Total nodes (individual)	5036 (63)	1047 (13)	1350 (17)	511 (7)
Gender				
Female	1261 (25)	193 (18)	76 (6)	0 (0)
Male	3775 (75)	854 (82)	1274 (94)	511 (100)
Race or ethnicity				
Non-Hispanic Black	2501 (50)	511 (49)	644 (48)	183 (36)
Non-Hispanic White	1157 (23)	268 (26)	327 (24)	114 (22)
Hispanic	1258 (25)	242 (23)	336 (25)	203 (40)
Non-Hispanic other	120 (2)	26 (2)	43 (3)	11 (2)
Age group (years)				
<25	829 (16)	247 (24)	448 (33)	171 (33)
25‐49	3282 (65)	689 (66)	815 (60)	312 (61)
50+	925 (18)	111 (11)	87 (6)	28 (5)
HIV risk group				
Hetero^b^	2022 (40)	276 (26)	119 (9)	5 (1)
IDU^c^	260 (5)	33 (3)	17 (1)	2 (0)
MMSC^d^	2602 (52)	698 (67)	1140 (84)	479 (94)
MMSC or IDU	152 (3)	40 (4)	74 (5)	25 (5)

a This line does not report any percentage and is, in fact, the label for each column, that is, the size in terms of total number of nodes we are describing.

b Hetero: heterosexual contact.

c IDU: injection drug use.

d MMSC: male-to-male sexual contact.

## Discussion

### Overview

Using the FDOH public dataset, we characterized DBPs for all documented people newly diagnosed with HIV in Florida between 2012 and 2022. A total of 37 DBPs were identified. The attempt to extract HIV behavioral risk groups via unsupervised clustering stratified data based on demographics only, as the behavioral component (ie, the mode of exposure) was not a discriminating feature even when deliberately overclustering. Therefore, a list of all combinations of variable categories was used to characterize DBP. DBPs were further used to examine area and county similarity across Florida and characterize contact networks. The results from these analyses can be used to inform future HIV prevention and intervention efforts by guiding public health professionals in making decisions on resource allocation and policy for their strategies to end the HIV epidemic. These findings can also potentially help public health workers in the field, allowing them to better understand trends in the HIV epidemic in their areas to identify and help community members most in need. Moreover, these findings show that contact tracing surveillance data can be used to investigate the dynamics of HIV transmission and support further research and development in this area for the creation of more informative models. Integrating these models into routine surveillance would enable regular communication of results to program staff and field workers, ensuring the continued effectiveness of current efforts while also allowing for strategies to be adapted in response to evolving needs.

The 6 largest networks (30+ nodes and 511 individuals) are almost exclusively composed of individuals with MMSC (ie, MSM). While the female, non-Hispanic Black, aged 25‐49 years, and hetero profile is the fourth largest DBP, none of the large networks involve females of any race or age. This may indicate that HIV transmission to women does not involve large contact networks with many potentially at-risk partners. If this is the case, then interventions that focus solely on increasing prevention efforts among those with multiple sexual partners may still leave many women vulnerable to HIV. The implementation of past prevention efforts has made similar missteps. For example, the original guidance for prescribing PrEP focused largely on and marketed toward MSM and included language that excluded at-risk women. As a consequence, PrEP awareness and uptake have remained low among at-risk women, although the guidelines have been updated to try and address this [27,28].

Those with MMSC comprise the dominant HIV risk group among the large networks identified. This matches national trends, with 67% of new HIV diagnoses in the United States in 2021 involving MMSC [29]. Additionally, in the United States, 79% of new HIV cases were among men, 40% were among Black individuals, 29% among Hispanic individuals, and 37% among those aged 25‐34 years [29], which closely mirrors the largest transmission networks that we identified in Florida. People with IDU may appear in the network, but no major network is characterized solely by IDU. This might be because IDU is not a common behavior, with less than 2% of the US population reporting IDU in 2018 [30], and ~4% in the extracted STARS DBPs report IDU. However, IDU is still a prominent means of HIV acquisition and transmission, accounting for about 10% of new cases [1], and should still be considered in prevention and intervention efforts. Hetero exposure is also not common in the identified networks, again indicating that exposure via hetero may occur mainly in small networks.

All of the large HIV transmission networks we identified involve individuals from multiple counties. Most of these networks were localized by region or represented multiple urban centers in the state. While neighboring counties are often grouped together into HIV coordination areas to address HIV, not all of these networks are contained in 1 area. Moreover, Florida has a high population who are seasonal residents, migrants, and transient tourists from all over the world. Close communication between county health officials is essential to address the HIV epidemic as these transmission networks extend beyond immediate neighboring counties. One of the goals of the HIV coordination areas is to help facilitate between-county communication, even though some areas are only 1 county (eg, areas 7B and 11A).

We identified 2 distinct clusters of HIV areas that divide the state roughly in half (Northern or Northwestern and Central or Southern) and 6 county clusters. Another level of categorization into 2 larger HIV area groups might also help increase communication between the counties that we identified as being within similar clusters. For example, the largest county cluster, represented counties within areas 6, 7A, 8A, 9, and 15, which are all in the Central or Southern Florida cluster, plus a county in area 14. Improving both interarea and intercounty communication could help improve information sharing between counties with similar DBPs, and information shared could include successful interventions and lessons learned from interventions that were less successful to improve future decision-making and resource allocation. Increasing discussions within these clusters could also improve information sharing between neighboring counties that happen to be in different HIV areas.

There are existing evidence-based and impactful strategies for addressing HIV, such as PrEP [16,17], postexposure prophylaxis [31], condoms [32], and syringe services programs [33,34]. Our findings support increasing the use of these and other prevention strategies in areas and among groups we identified as having high transmission. For example, our findings identified those with MMSC in Miami-Dade as a high-transmission contact network, and past research has found that there is low uptake of PrEP among MSM in Miami-Dade [35]. Additionally, testing efforts among high-risk groups can be increased in geographic areas and among groups identified in this study. Our findings can enable focused and efficient resource allocation, prioritizing efforts and funding for groups at high HIV risk and areas with increased HIV transmission.

When characterizing DBPs in identified contact networks, we intentionally only include these DBPs at an aggregated level due to ethical concerns. Moreover, we would like to emphasize that many of the groups and behaviors that we have identified in these clusters are groups that already experience high levels of stigma and discrimination. The goal of this work is not to contribute to this existing stigma, but to highlight areas of need and groups who might need targeted intervention to reduce their risk of HIV. There is a continuous need to conduct ethical discussions to avoid the possible harm of advanced methodologies to vulnerable groups, especially in the context of HIV stigmatization [22].

Our results should be interpreted in consideration of some limitations. We removed STARS records without linked partners or who had missing or inconsistent records, so our network analyses were conducted on a subset of STARS data. The extracted STARS DBPs have a high correlation with DBPs identified from the Florida public dataset [13], indicating good representativeness. The data are retrospective, which could limit their usefulness for public health workers in the field if they simply confirm what they already know about the HIV epidemic in their area rather than provide new information. However, these findings support further investigation and application of contact tracing surveillance data to provide a more up-to-date and accurate understanding of transmission dynamics, allowing more targeted and impactful prevention and intervention efforts. A final consideration is that extracting DBPs from the FDOH public dataset via unsupervised clustering leads to clusters solely characterized by nonbehavioral components, such as gender and race or ethnicity, therefore missing the mode of exposure, a key feature to explore the HIV contact networks. A primary strength of this study is our public health data, with high representativeness of people with HIV in the last 10 years in Florida.

### Conclusions

In our contact network analysis, we identified several distinct risk groups (young MSM, non-Hispanic Black MSM, Hispanic MSM, Hispanic women, and people with IDU) and clusters of transmission in Florida (divided generally into Northern or Northwestern and Central or Southern, with some similarities identified at the county level). These results can be used to inform targeted interventions aimed at reducing HIV diagnoses in the state. These groups we identified are already recognized as having a disproportionately high risk of HIV in Florida and nationwide, and our findings have shown areas in the state where these disparities might be exacerbated. Inequitable distribution of the burden of HIV among these groups contributes to existing health disparities and shows that the HIV epidemic will not be ended by a one-size-fits-all approach. Network analysis can help state and county health departments not only identify disparities and areas or groups with high transmission but also make the best use of their HIV intervention and prevention resources to help their community members.

## Supplementary material

10.2196/65573 Multimedia Appendix 1 Similarity between DBPs from an MCA. DBP: demographic-behavioral profile; MCA: multiple correspondence analysis.

## Abbreviations

DBP: demographic-behavioral profile
DIS: disease intervention specialist
EHE: Ending the HIV Epidemic
FDOH: Florida Department of Health
hetero: heterosexual contact
HIPAA: Health Insurance Portability and Accountability Act
IDU: injection drug use
IRB: institutional review board
MCA: multiple correspondence analysis
MMSC: male-to-male sexual contact
MSM: men who have sex with men
PrEP: pre-exposure prophylaxis
PRISM: Patient Reporting Investigation Surveillance Manager
STARS: Surveillance Tools and Reporting System
