# Supplementary material for: Behavioral and Demographic Profiles of HIV Transmission and Exposure Networks in Florida: Network Analysis of HIV Contact Tracing Data
Source: JMIR Public Health Surveill. 2025 Jun 25;11:e65573. doi: 10.2196/65573 (PMC12242702; doi:10.2196/65573)

**Figure S1.** Similarity between DBPs from an MCA. DBP: demographic-behavioral profile; MCA: multiple correspondence analysis.


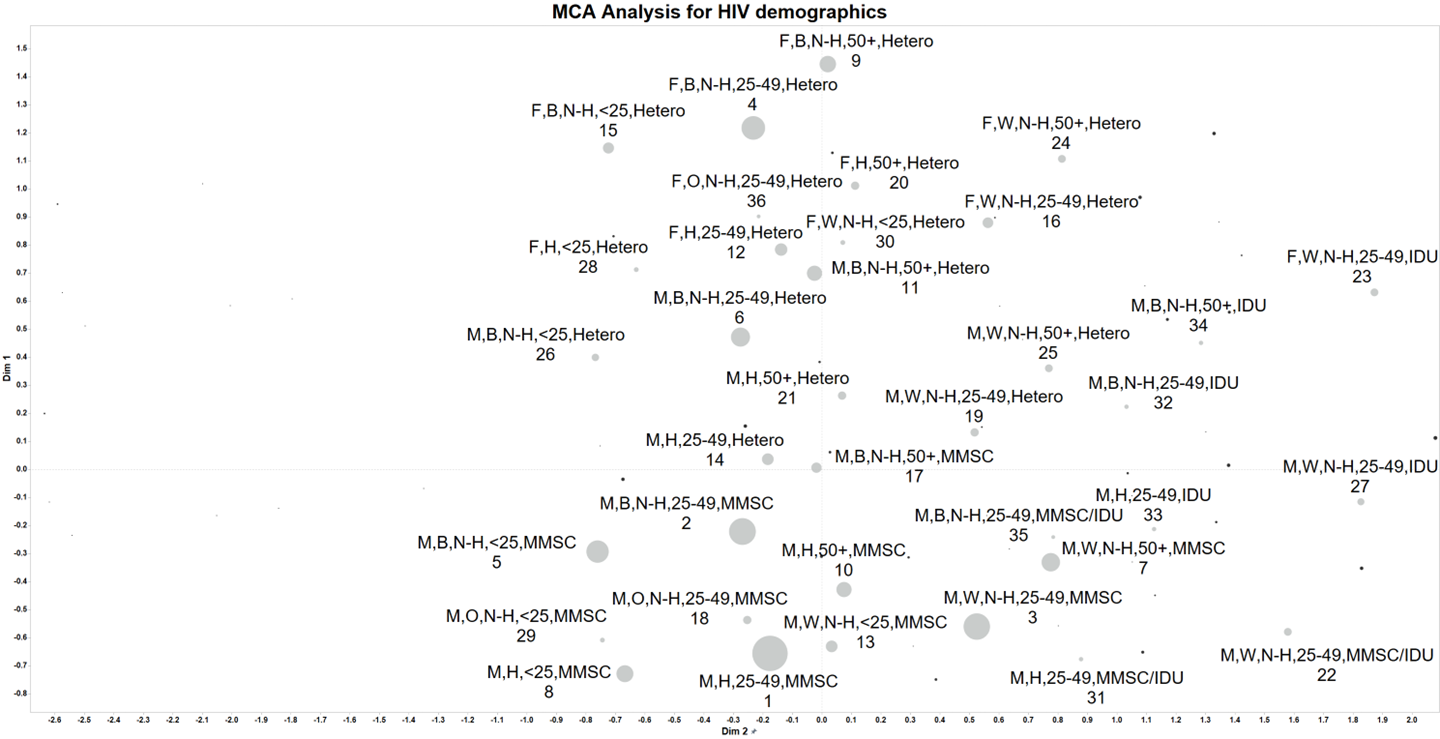

Supplement: Multimedia Appendix 1 [file publichealth-v11-e65573-s001.docx]
